# Alum in Typhoid Fever

**Published:** 1888-06

**Authors:** 


**Alum in Typhoid Fever.**—Dr. Paoletti has treated sixty cases of typhoid fever, with excellent results, with crude alum alone. He remarks that this drug has formerly been used only as a styptic and an astringent, but now that its antiseptic properties have been recognized it is clearly indicated as a remedy for abnomal fermentations in the intestinal canal.—*British Medical Journal, February, 1888.*

